# Supplementary material for: COVID‐19 pandemic‐related lockdown: response time is more important than its strictness
Source: EMBO Mol Med. 2020 Oct 19;12(11):e13171. doi: 10.15252/emmm.202013171 (PMC7645374; doi:10.15252/emmm.202013171)
Supplement: Supplementary file 1 — Appendix [file EMMM-12-e13171-s001.pdf]

# COVID-19 pandemic-related lockdown: response time is more important than its strictness

# Appendix

Gil Loewenthal[1, *], Shiran Abadi[2, *], Oren Avram[1, *], Keren Halabi[2, *], Noa Ecker[1, *], Natan Nagar[1], Itay Mayrose[2, †], and Tal Pupko[1, †]

[1] The Shmunis School of Biomedicine and Cancer Research, George S. Wise Faculty of Life Sciences, Tel Aviv University, Tel Aviv 69978, Israel.

[2] School of Plant Sciences and Food Security, George S. Wise Faculty of Life Sciences, Tel Aviv University, Tel Aviv 69978, Israel.

<u>**Figures**</u> **Pages**

Appendix Figure S1. Mobility and number of daily deaths as a function of time 2-4

Appendix Figure S2. Time points for synchronizing the death model and the mobility model 5-7

Appendix Figure S3. A semi-logarithmic scatter plot of the Aug-20 *COVID-19 Mortality Probability* and τ in 61 countries 8

Appendix Figure S4. A semi-logarithmic scatter plot of the Aug-20 *COVID-19 Mortality Probability* and τ for the states within the United States 9

Appendix Figure S5. A semi-logarithmic scatter plot of the Aug-20 *COVID-19 Infection Probability* and τ 10

Appendix Figure S6. Mobility percentages in Italy at lockdown declarations 11

<u>**Tables**</u>

Appendix Table S1. Inferred values for mobility model parameters for all 37 OECD countries 12

Appendix Table S2. Features extracted from the mobility model 13

Appendix Table S3. Inferred values for the death model parameters for all 37 OECD countries 14

Appendix Table S4. Regression analysis 15

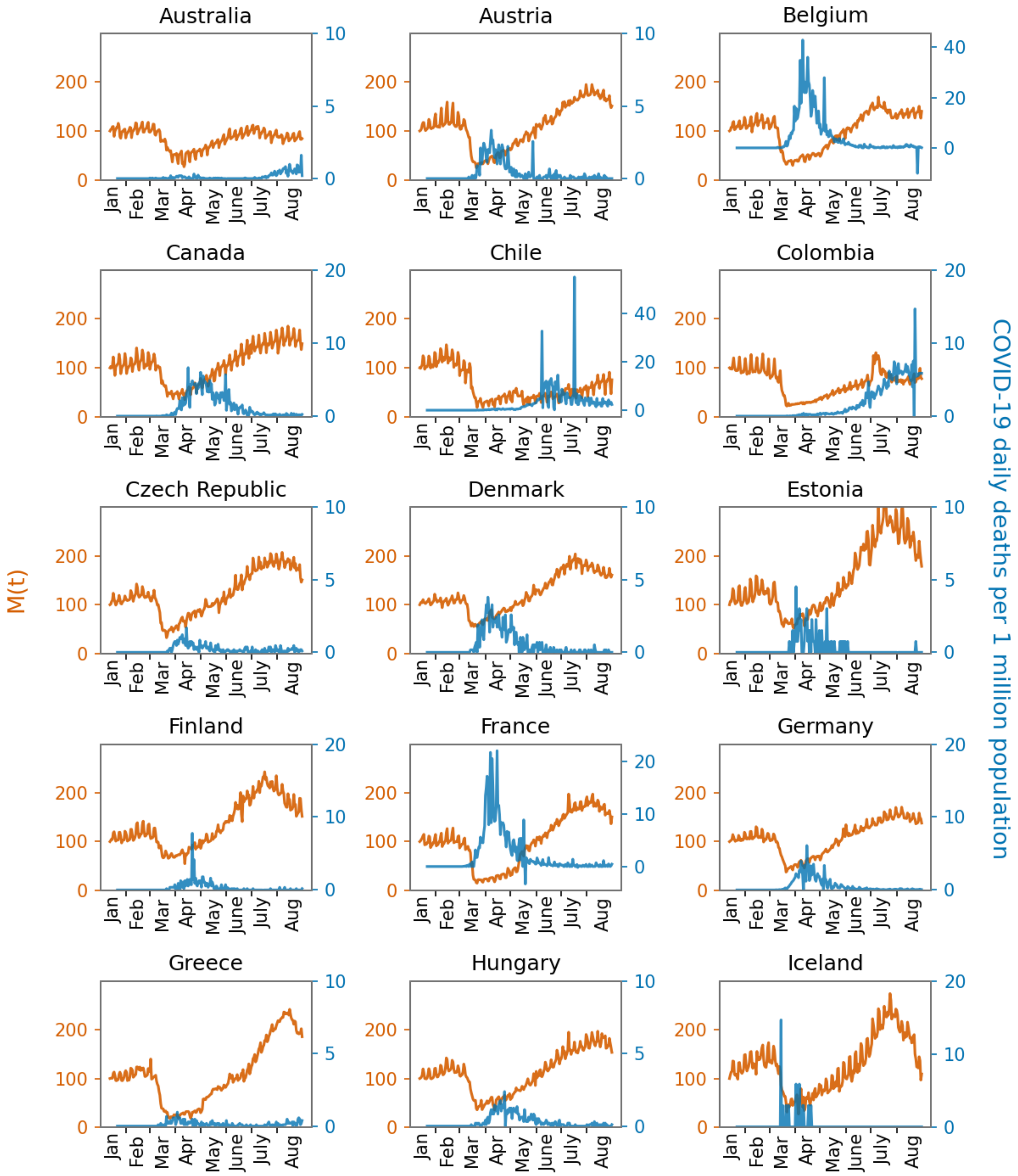
Australia
Austria
Belgium
Canada
Chile
Colombia
Czech Republic
Denmark
Estonia
Finland
France
Germany
Greece
Hungary
Iceland
M(t)
COVID-19 daily deaths per 1 million population
Jan
Feb
Mar
Apr
May
June
July
Aug

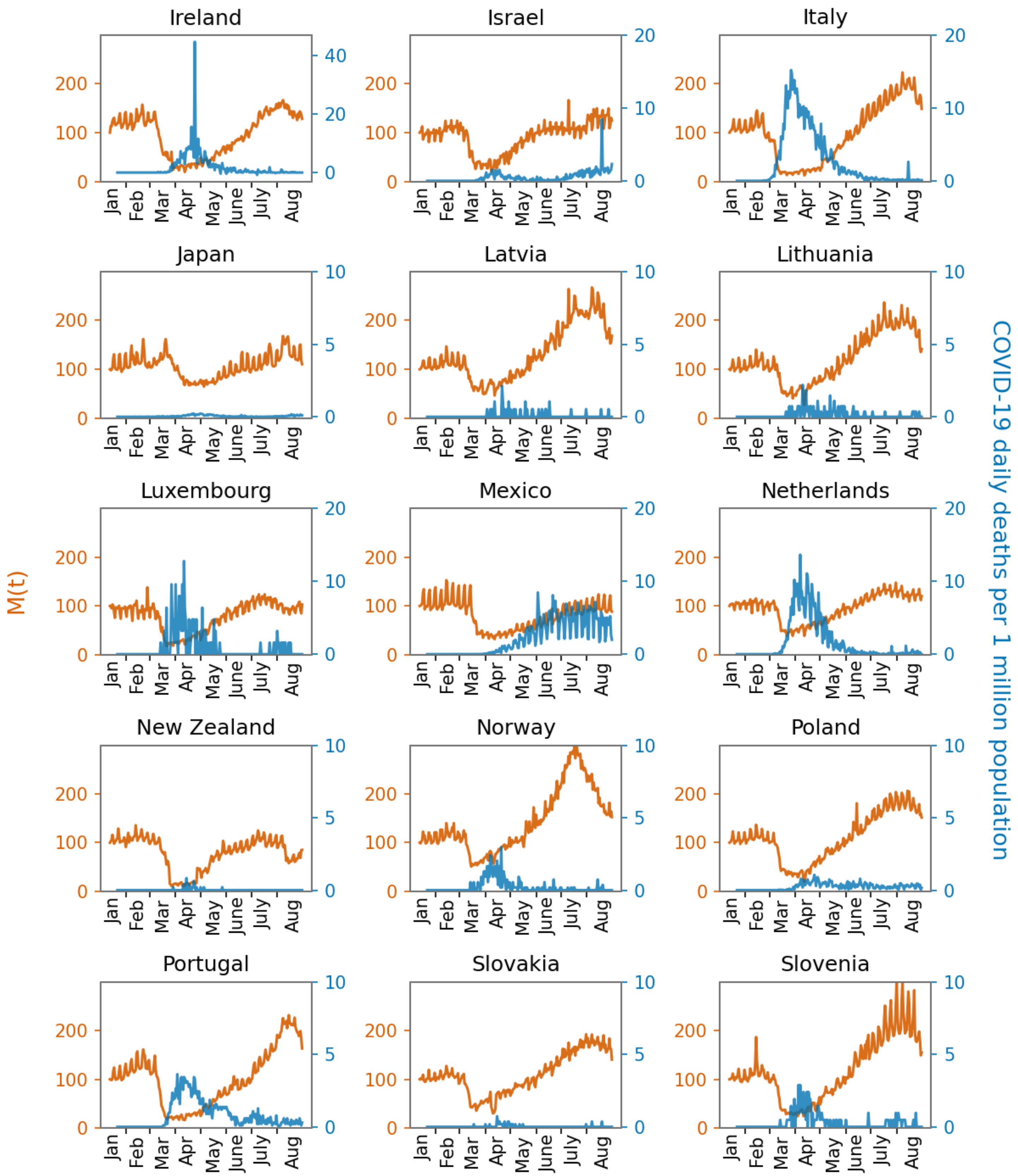
Ireland
Israel
Italy
Japan
Latvia
Lithuania
Luxembourg
Mexico
Netherlands
New Zealand
Norway
Poland
Portugal
Slovakia
Slovenia
M(t)
COVID-19 daily deaths per 1 million population
Jan
Feb
Mar
Apr
May
June
July
Aug

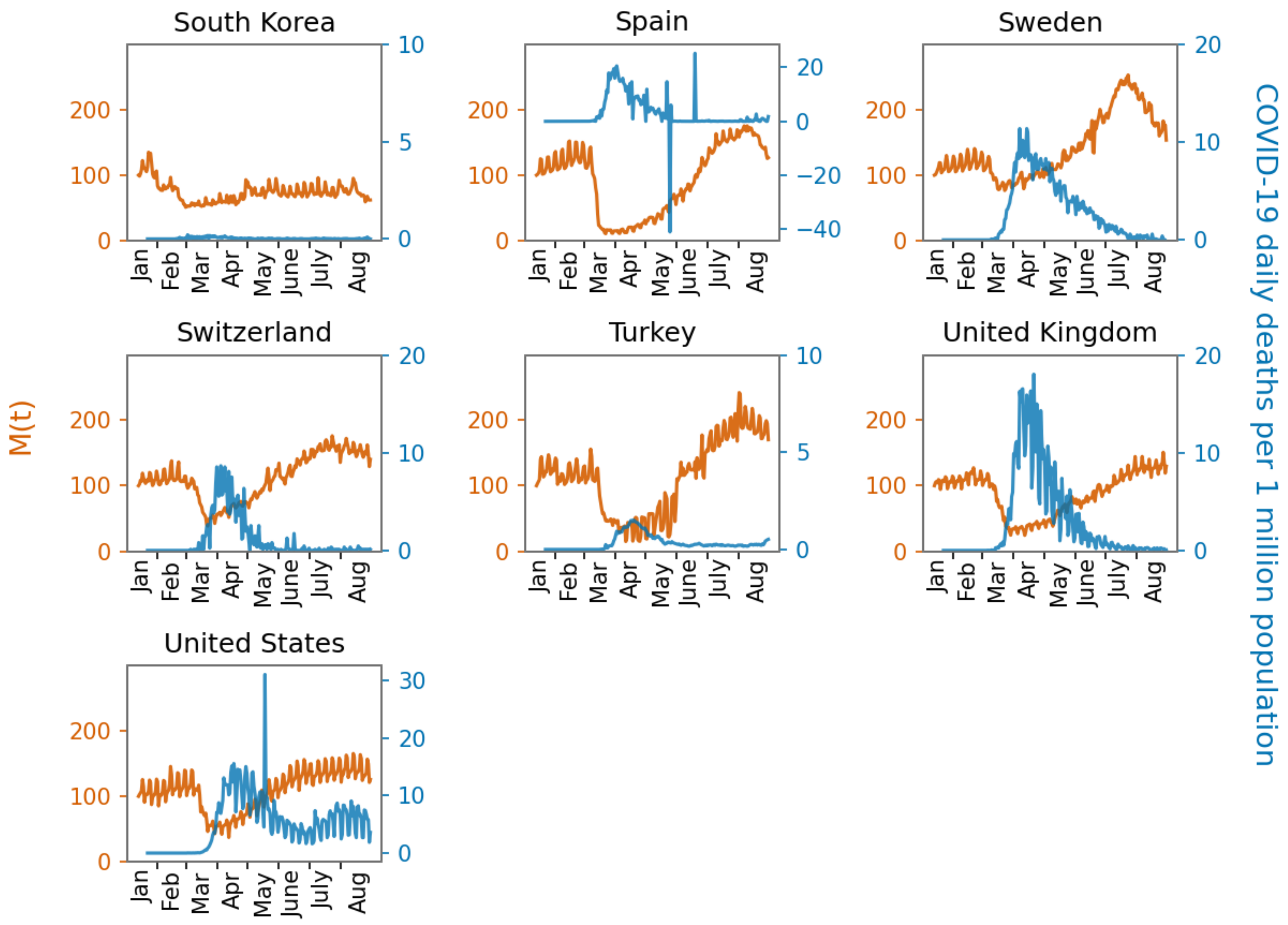


**Appendix Figure S1. Mobility and number of daily deaths as a function of time.** The orange lines represent the daily mobility in percentage from January 13 to August 31, 2020, compared to 100% set on January 13 as the baseline (left y axis). The blue lines represent the daily deaths, from January 22 to August 31, 2020, relative to 1-million population size (right y axis). Graphs are shown for all 37 OECD countries, ordered alphabetically.

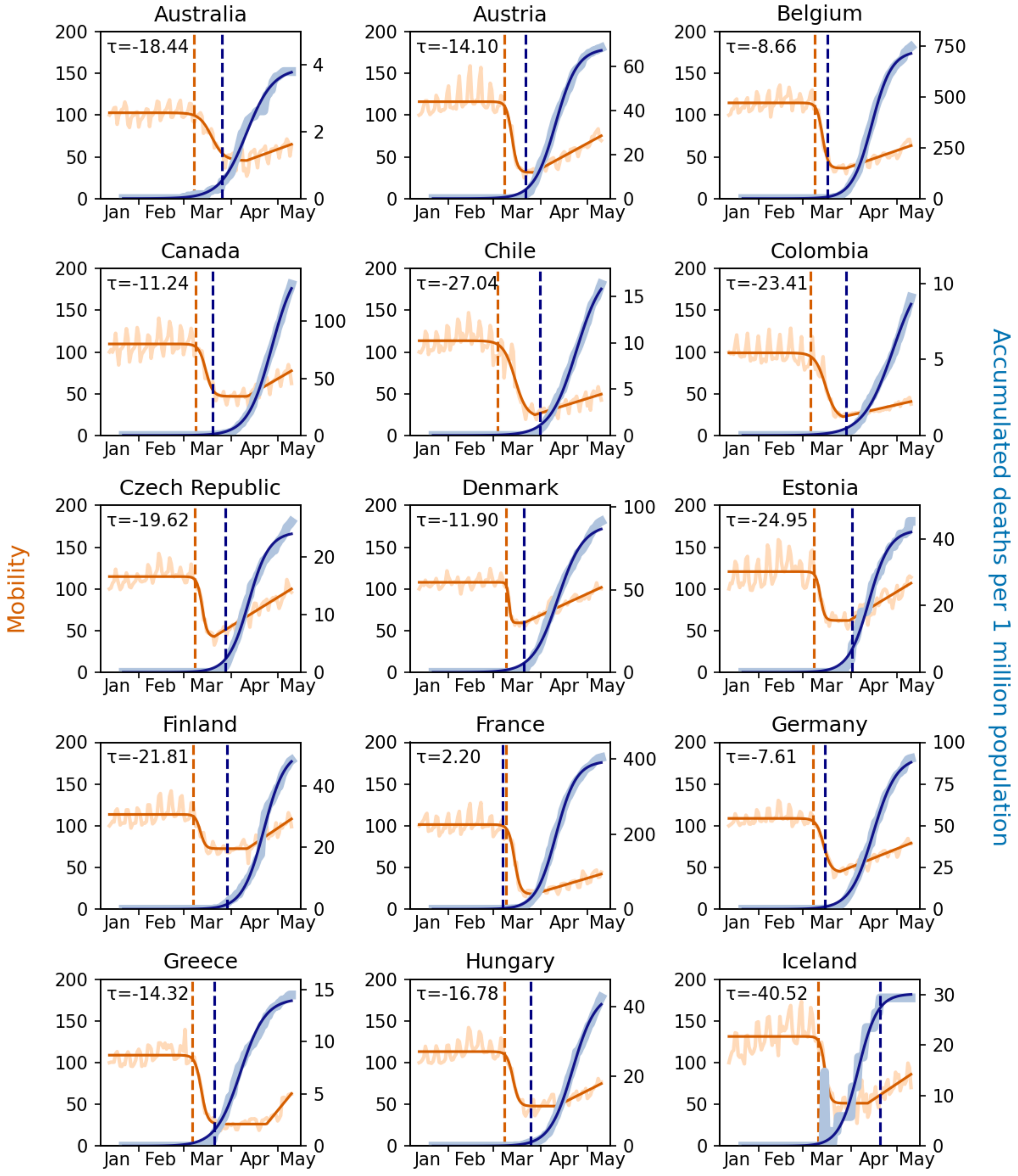
Australia
τ=-18.44
Austria
τ=-14.10
Belgium
τ=-8.66
Canada
τ=-11.24
Chile
τ=-27.04
Colombia
τ=-23.41
Czech Republic
τ=-19.62
Denmark
τ=-11.90
Estonia
τ=-24.95
Finland
τ=-21.81
France
τ=2.20
Germany
τ=-7.61
Greece
τ=-14.32
Hungary
τ=-16.78
Iceland
τ=-40.52
Jan Feb Mar Apr May
Mobility
Accumulated deaths per 1 million population

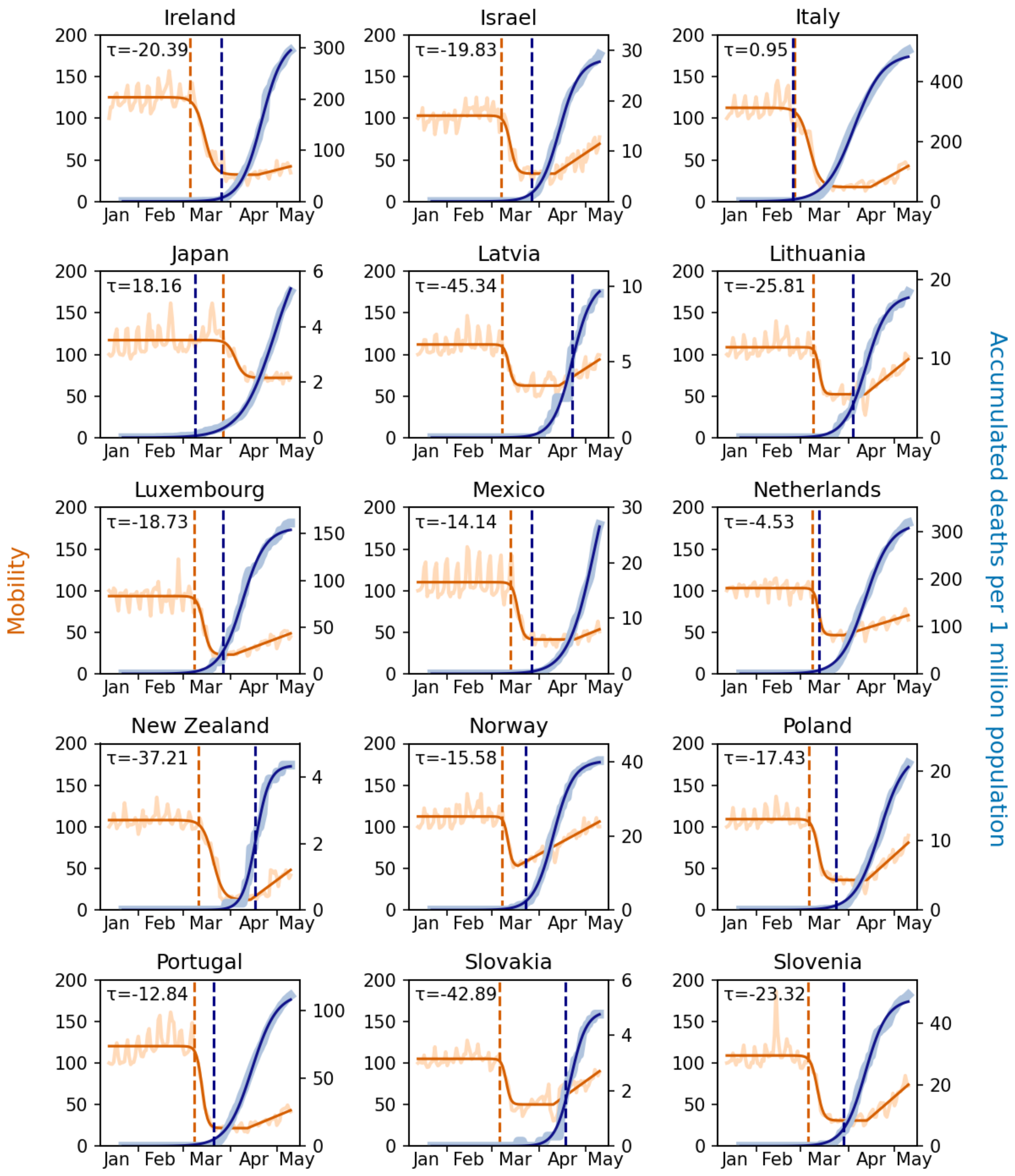
Ireland
τ=-20.39
Israel
τ=-19.83
Italy
τ=0.95
Japan
τ=18.16
Latvia
τ=-45.34
Lithuania
τ=-25.81
Luxembourg
τ=-18.73
Mexico
τ=-14.14
Netherlands
τ=-4.53
New Zealand
τ=-37.21
Norway
τ=-15.58
Poland
τ=-17.43
Portugal
τ=-12.84
Slovakia
τ=-42.89
Slovenia
τ=-23.32
Jan Feb Mar Apr May
Mobility
Accumulated deaths per 1 million population

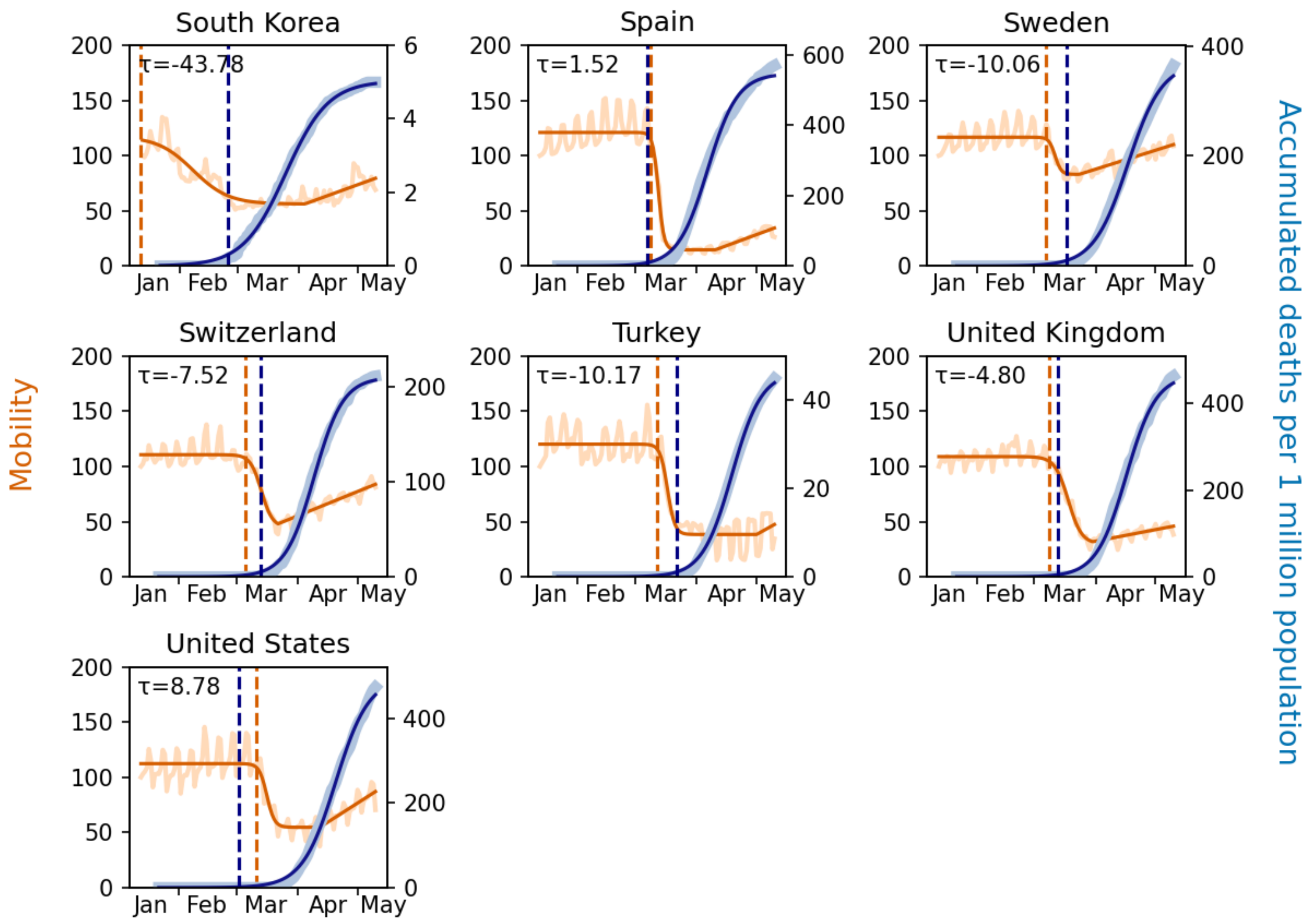


**Appendix Figure S2. Time points for synchronizing the death model and the mobility model.** Data are shown for 37 OECD countries, ordered alphabetically. The dark orange plots represent the mobility model, $\widehat{M}(t)$, fitted to the mobility data, $M(t)$ from January 13 to May 10, 2020 (light orange; left y axis). The dashed vertical orange line represents the *social distancing start time*, according to the mobility model. The dark blue plots represent the death model, $\widehat{D}(t)$, fitted to the accumulated death data, $D(t)$ from January 22 to May 10, 2020 (light blue; right y axis) of the countries. The dashed vertical blue lines represent the day ten deaths were documented. $\tau$ represents the difference between the orange and the blue vertical lines and corresponds to the response time (values are denoted in each panel). τ is negative for countries that initiated social distancing before ten deaths were documented.

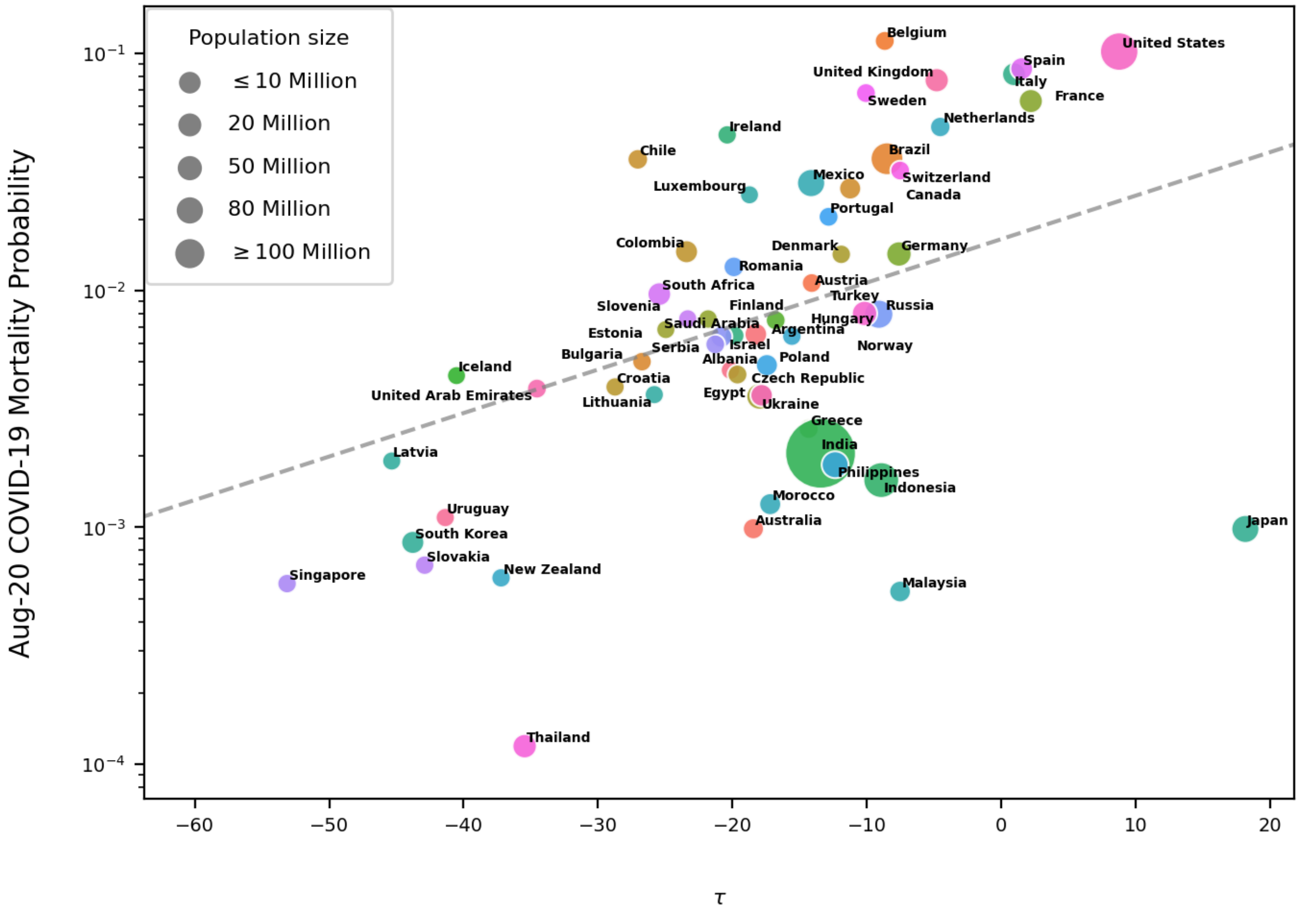


**Appendix Figure S3. A semi-logarithmic scatter plot of the AUG-20 *COVID-19 Mortality Probability* and τ in 61 countries.** The x axis represents τ, the difference between the *social distancing start time* and the day in which the first ten deaths were recorded for the respective country (intuitively, the response time). The y axis represents the *COVID-19 Mortality Probability* in a logarithmic scale. Among the 63 for which mobility and death data exist, Cambodia, Macau, and Taiwan did not reach ten deaths by August 31, 2020. Hong Kong and Vietnam comply with the regression line but are excluded for visualization due to extreme values ($\tau =$-176.52 and -138.144 and $\text{Aug} - 20\ COVID - 19\ Mortality\ Probability = 4e - 4$ and $7e - 6$ for Hong Kong and Vietnam, respectively). Dot sizes are proportional to population sizes. Pearson $r^2 = 0.39$ ($P\ value = 1e - 7$) when including Japan and $r^2 = 0.44$ ($P\ value = 1e - 8$) excluding Japan ($P\ values < 1e - 7$). The dashed line corresponds to the fitted regression, excluding Japan: $\log(\text{Aug} - 20\ COVID - 19\ Mortality\ Probability) = 0.018\tau - 1.78$ .

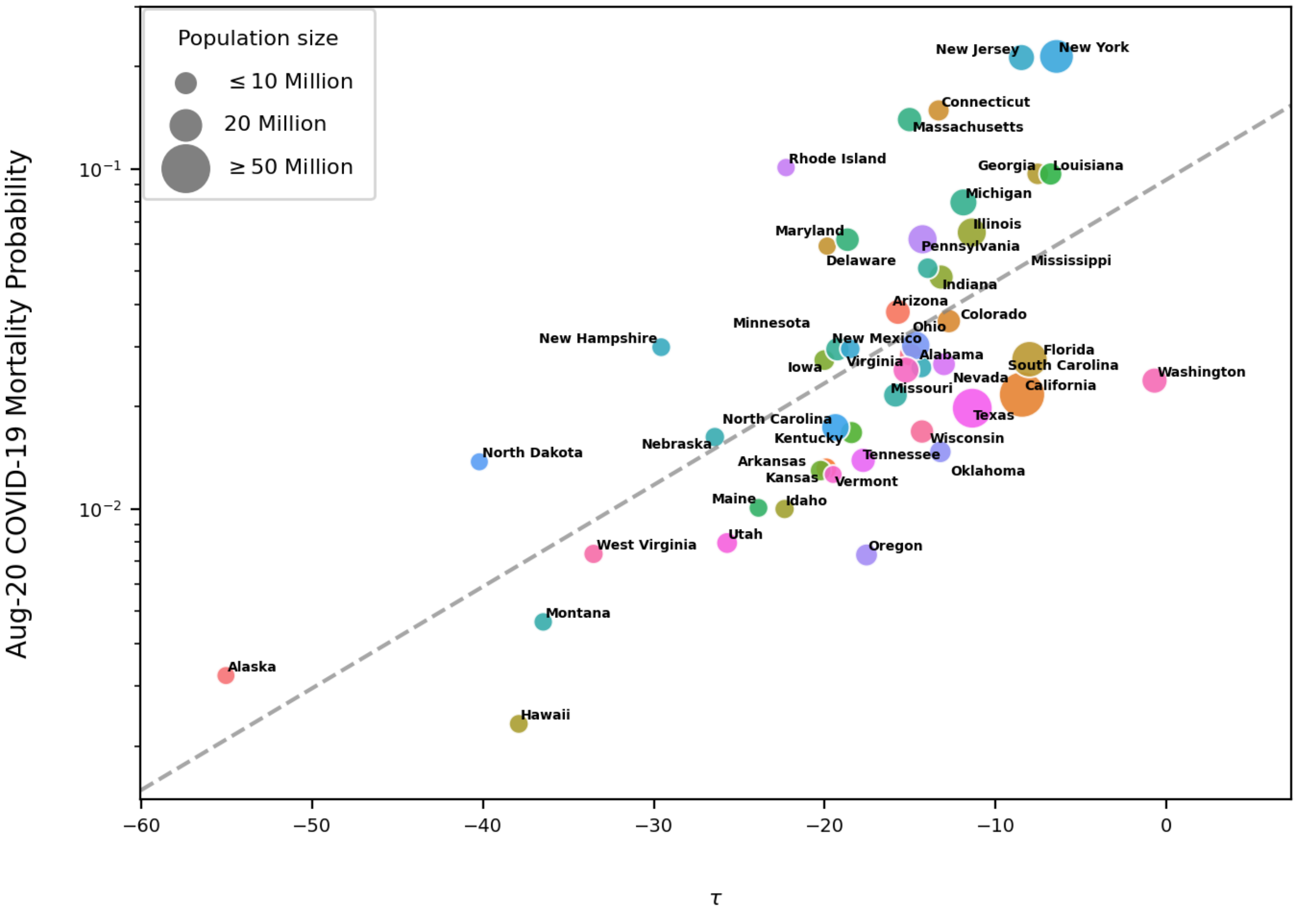


**Appendix Figure S4. A semi-logarithmic scatter plot of the AUG-20 *COVID-19 Mortality Probability* and τ for states within the United States.** The x axis represents τ, the difference between the *social distancing start time* and the day in which the first ten deaths were recorded for the respective country (intuitively, the response time). The y axis represents the *COVID-19 Mortality Probability* in a logarithmic scale. Dot sizes are proportional to population sizes. Pearson $r^2 = 0.44$ ($P\ value = 3e - 7$). Wyoming and South Dakota were excluded due to insufficient data. The dashed line corresponds to the fitted regression, $\log\left(\mathrm{AUG} - 20\ COVID - 19\ Mortality\ Probability\ \right) = 0.03\tau - 1.03$ .

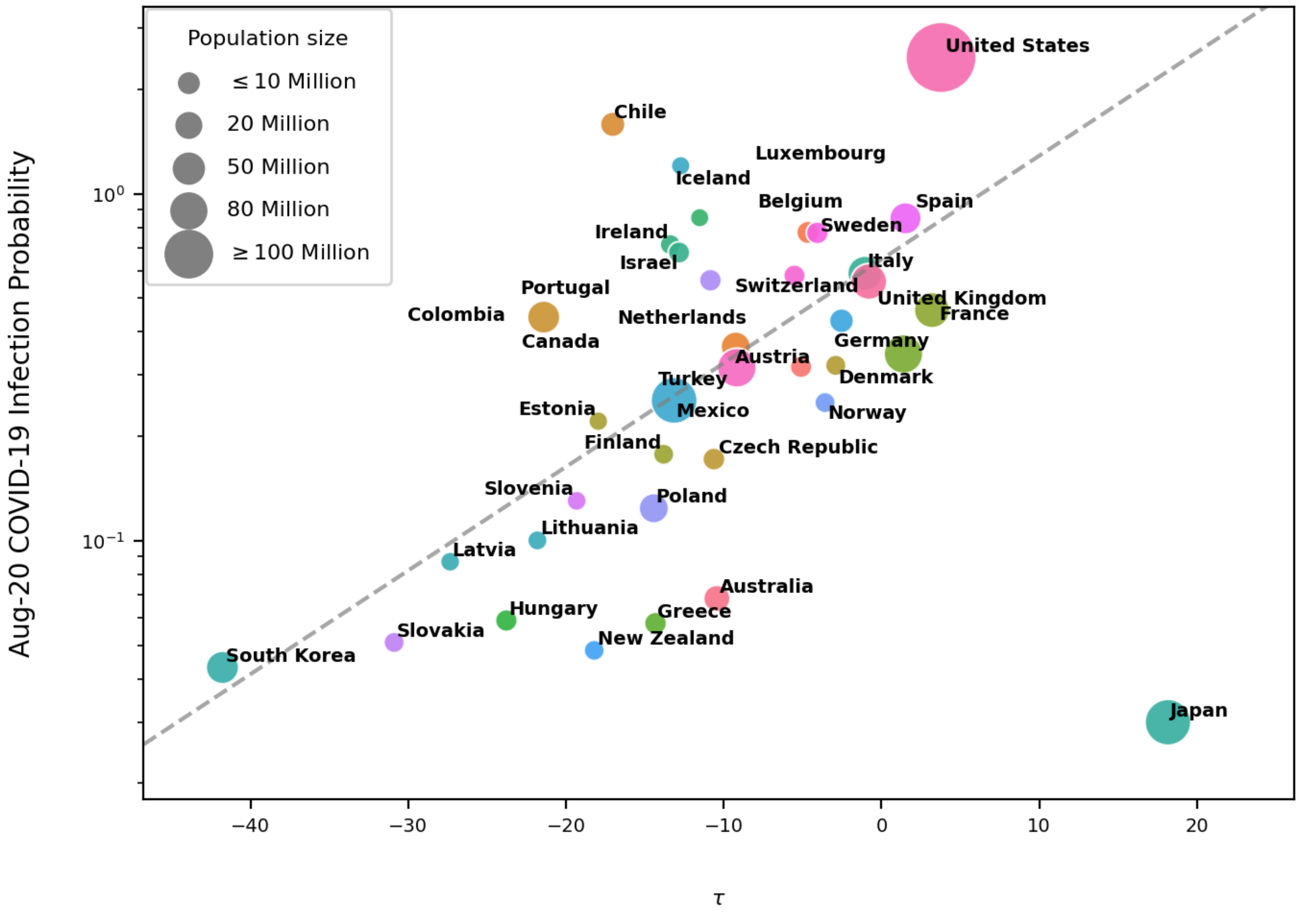


**Appendix Figure S5. A semi-logarithmic scatter plot of the AUG-20 *COVID-19 Infection Probability* and τ.** The x axis represents $\tau$, the difference between the *social distancing start time* and the day in which the first 500 confirmed cases were recorded for the respective country (intuitively, the response time). The y axis represents the AUG-20 *COVID-19 Infection Probability*, which is the number of confirmed cases in August 31, 2020, divided by the population size (in a logarithmic scale). Dot sizes are proportional to population sizes. Pearson $r^2 = 0.16\,(P\ value = 1e-2)$ when including Japan and $r^2 = 0.42\,(P\ value = 2e-5)$ excluding Japan. The dashed line corresponds to the fitted regression, excluding Japan, $\log(\mathrm{AUG}-20\ COVID-19\ Infection\ Probability) = 0.03\,\tau - 0.19$ .

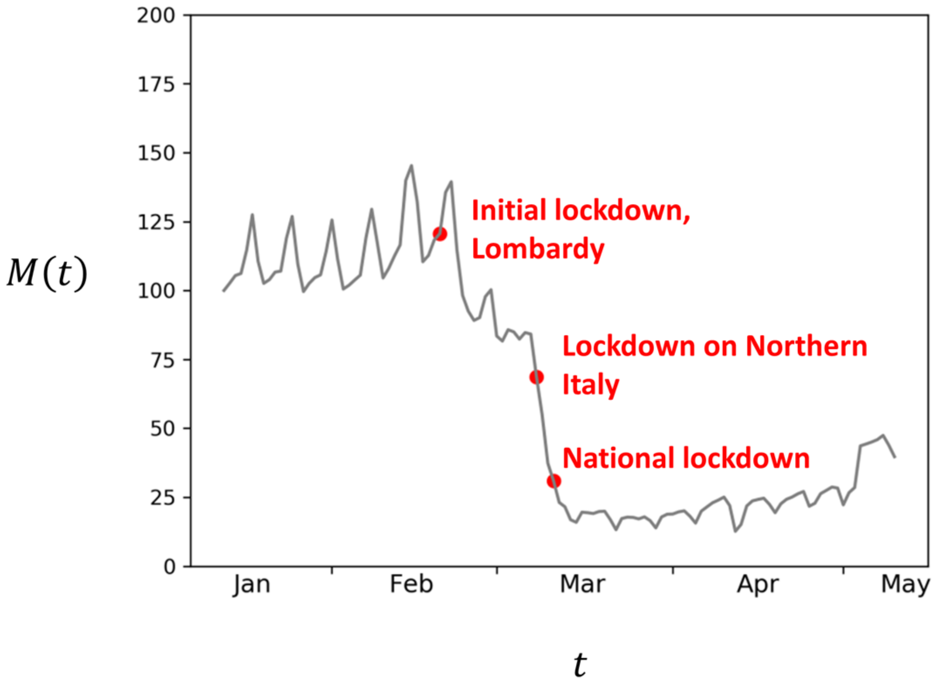


**Appendix Figure S6. Mobility percentages in Italy at lockdown declarations.** The x axis represents the time in days and the y axis represents the mobility data, $M(t)$, in Italy. Three lockdowns were declared in Italy: first in Lombardy and Veneto regions on February 20. On March 8 the lockdown expanded to all of Northern Italy, and on March 11 to a nationwide lockdown.

**Appendix Table S1. Inferred values for the mobility model parameters for all 37 OECD countries.**

| Country | $L$ | $b$ | $t^0$ | $t^1$ | $k$ | $a$ | Pearson $r^2$ | $P\ value$ |
|---|---|---|---|---|---|---|---|---|
| Australia | 57.36 | 45.43 | 66.26 | 89.00 | -0.25 | 0.67 | 0.90 | 4E-61 |
| Austria | 84.74 | 31.32 | 60.12 | 74.29 | -0.56 | 1.00 | 0.92 | 3E-66 |
| Belgium | 78.22 | 36.44 | 60.57 | 76.00 | -0.56 | 0.64 | 0.96 | 1E-81 |
| Canada | 62.78 | 46.85 | 62.44 | 89.43 | -0.44 | 1.07 | 0.89 | 8E-58 |
| Chile | 91.65 | 21.87 | 62.09 | 75.00 | -0.26 | 0.57 | 0.92 | 2E-66 |
| Colombia | 79.21 | 19.78 | 62.58 | 74.00 | -0.29 | 0.41 | 0.93 | 1E-70 |
| Czech Republic | 72.34 | 42.39 | 60.09 | 68.00 | -0.62 | 1.14 | 0.92 | 3E-65 |
| Denmark | 48.55 | 59.29 | 58.25 | 67.83 | -1.37 | 0.85 | 0.93 | 2E-68 |
| Estonia | 58.51 | 62.15 | 59.20 | 78.00 | -0.71 | 1.12 | 0.78 | 3E-40 |
| Finland | 40.96 | 72.62 | 58.96 | 89.36 | -0.62 | 1.24 | 0.80 | 5E-43 |
| France | 83.28 | 18.16 | 61.75 | 74.65 | -0.53 | 0.55 | 0.96 | 4E-83 |
| Germany | 64.39 | 44.44 | 61.42 | 72.00 | -0.42 | 0.74 | 0.95 | 3E-77 |
| Greece | 82.54 | 26.45 | 59.52 | 101.86 | -0.50 | 2.26 | 0.96 | 3E-85 |
| Hungary | 65.30 | 47.96 | 60.84 | 89.00 | -0.52 | 0.93 | 0.92 | 1E-65 |
| Iceland | 79.96 | 51.45 | 62.23 | 90.00 | -0.62 | 1.24 | 0.86 | 5E-51 |
| Ireland | 92.94 | 32.30 | 62.07 | 96.87 | -0.31 | 0.47 | 0.95 | 6E-78 |
| Israel | 69.69 | 33.51 | 60.06 | 89.00 | -0.50 | 1.23 | 0.91 | 7E-64 |
| Italy | 95.22 | 17.38 | 53.79 | 93.44 | -0.30 | 1.03 | 0.96 | 7E-81 |
| Japan | 45.09 | 72.07 | 82.17 | 89.00 | -0.39 | 1.00 | 0.71 | 1E-33 |
| Latvia | 49.39 | 62.59 | 59.15 | 91.72 | -0.66 | 1.19 | 0.84 | 6E-49 |
| Lithuania | 56.52 | 52.17 | 60.13 | 90.00 | -0.75 | 1.51 | 0.88 | 1E-55 |
| Luxembourg | 70.34 | 23.11 | 61.45 | 81.06 | -0.48 | 0.69 | 0.93 | 6E-70 |
| Mexico | 68.82 | 41.34 | 65.03 | 101.68 | -0.57 | 0.75 | 0.85 | 1E-50 |
| Netherlands | 56.51 | 46.53 | 59.62 | 76.00 | -0.71 | 0.57 | 0.95 | 5E-76 |
| New Zealand | 95.78 | 12.31 | 67.57 | 91.93 | -0.30 | 1.37 | 0.96 | 6E-86 |
| Norway | 59.83 | 52.61 | 58.55 | 65.00 | -0.71 | 1.00 | 0.86 | 2E-51 |
| Poland | 73.13 | 36.12 | 59.29 | 91.00 | -0.51 | 1.66 | 0.95 | 7E-79 |
| Portugal | 98.53 | 21.75 | 60.38 | 90.00 | -0.56 | 0.77 | 0.94 | 8E-73 |
| South Korea | 61.15 | 56.14 | 25.66 | 82.26 | -0.11 | 0.65 | 0.82 | 5E-45 |
| Slovakia | 54.87 | 50.17 | 57.39 | 88.00 | -0.69 | 1.33 | 0.89 | 9E-58 |
| Slovenia | 78.13 | 30.89 | 58.76 | 91.14 | -0.48 | 1.60 | 0.91 | 7E-64 |
| Spain | 106.28 | 14.70 | 59.62 | 88.29 | -0.72 | 0.67 | 0.96 | 2E-82 |
| Sweden | 33.67 | 83.00 | 58.45 | 70.57 | -0.65 | 0.57 | 0.70 | 1E-32 |
| Switzerland | 65.63 | 44.97 | 60.64 | 69.00 | -0.36 | 0.73 | 0.92 | 2E-67 |
| Turkey | 81.77 | 38.38 | 64.20 | 109.04 | -0.55 | 1.01 | 0.90 | 2E-60 |
| United Kingdom | 79.06 | 29.80 | 65.09 | 78.00 | -0.30 | 0.35 | 0.97 | 4E-92 |
| United States | 57.63 | 54.67 | 63.37 | 90.00 | -0.53 | 1.15 | 0.84 | 5E-48 |
| **Mean** | **70.26** | **41.44** | **60.51** | **84.36** | **-0.53** | **0.97** | **0.90** | |
| **STD** | **17.11** | **17.34** | **7.32** | **10.63** | **0.21** | **0.40** | **0.07** | |

**Appendix Table S2. Features extracted from the mobility model.**

| Country | *Social distancing start time* | *Minimal mobility time point* | *Drop duration* | *Lockdown strictness* | *Lockdown duration* | *Lockdown release day* | *Lockdown release rate* |
|---|---|---|---|---|---|---|---|
| Australia | 54.56 | 77.95 | 23.39 | 55.70 | 11.05 | 16.00 | 0.67 |
| Austria | 54.90 | 65.34 | 10.44 | 73.00 | 8.95 | 5.29 | 1.00 |
| Belgium | 55.34 | 65.81 | 10.47 | 68.21 | 10.19 | 12.00 | 0.64 |
| Canada | 55.76 | 69.11 | 13.35 | 57.27 | 20.32 | 22.43 | 1.07 |
| Chile | 50.96 | 73.22 | 22.26 | 78.71 | 1.78 | -3.00 | 0.57 |
| Colombia | 52.59 | 72.58 | 19.99 | 77.93 | 1.42 | -2.00 | 0.41 |
| Czech Republic | 55.38 | 64.81 | 9.44 | 62.77 | 3.19 | -7.00 | 1.14 |
| Denmark | 56.10 | 60.40 | 4.30 | 45.02 | 7.44 | -0.17 | 0.85 |
| Estonia | 55.05 | 63.35 | 8.30 | 48.49 | 14.65 | -2.00 | 1.12 |
| Finland | 54.19 | 63.73 | 9.54 | 36.06 | 25.63 | 13.36 | 1.24 |
| France | 56.20 | 67.30 | 11.10 | 82.00 | 7.35 | 20.65 | 0.55 |
| Germany | 54.39 | 68.45 | 14.06 | 58.76 | 3.55 | 10.00 | 0.74 |
| Greece | 53.68 | 65.36 | 11.69 | 75.73 | 36.50 | 33.86 | 2.26 |
| Hungary | 55.22 | 66.46 | 11.23 | 57.65 | 22.54 | 17.00 | 0.93 |
| Iceland | 57.48 | 66.98 | 9.50 | 60.85 | 23.02 | -8.00 | 1.24 |
| Ireland | 52.61 | 71.52 | 18.91 | 74.21 | 25.35 | 23.87 | 0.47 |
| Israel | 54.17 | 65.95 | 11.78 | 67.53 | 23.05 | 15.00 | 1.23 |
| Italy | 43.95 | 63.64 | 19.69 | 84.57 | 29.80 | 50.44 | 1.03 |
| Japan | 74.68 | 89.66 | 14.98 | 37.56 | -0.66 | 33.00 | 1.00 |
| Latvia | 54.66 | 63.63 | 8.98 | 44.11 | 28.09 | -8.28 | 1.19 |
| Lithuania | 56.19 | 64.07 | 7.88 | 52.00 | 25.93 | 8.00 | 1.51 |
| Luxembourg | 55.27 | 67.62 | 12.35 | 75.26 | 13.44 | 7.06 | 0.69 |
| Mexico | 59.86 | 70.21 | 10.36 | 62.48 | 31.47 | 27.68 | 0.75 |
| Netherlands | 55.47 | 63.76 | 8.30 | 54.84 | 12.24 | 16.00 | 0.57 |
| New Zealand | 57.79 | 77.36 | 19.57 | 88.54 | 14.57 | -3.07 | 1.37 |
| Norway | 54.42 | 62.68 | 8.26 | 52.93 | 2.32 | -5.00 | 1.00 |
| Poland | 53.57 | 65.01 | 11.45 | 66.94 | 25.99 | 20.00 | 1.66 |
| Portugal | 55.16 | 65.60 | 10.44 | 81.92 | 24.40 | 22.00 | 0.77 |
| Slovakia | 53.11 | 61.68 | 8.58 | 52.24 | 26.32 | -8.00 | 1.33 |
| Slovenia | 52.68 | 64.84 | 12.16 | 71.67 | 26.30 | 15.14 | 1.60 |
| South Korea | -0.78 | 52.09 | 52.87 | 52.08 | 30.17 | 39.26 | 0.65 |
| Spain | 55.52 | 63.72 | 8.20 | 87.85 | 24.56 | 34.29 | 0.67 |
| Sweden | 53.94 | 62.97 | 9.04 | 28.85 | 7.60 | 5.57 | 0.57 |
| Switzerland | 52.48 | 68.80 | 16.31 | 57.74 | 0.20 | 9.00 | 0.73 |
| Turkey | 58.83 | 69.56 | 10.73 | 68.06 | 39.48 | 40.04 | 1.01 |
| United Kingdom | 55.20 | 74.97 | 19.77 | 71.18 | 3.03 | 18.00 | 0.35 |
| United States | 57.78 | 68.96 | 11.18 | 51.32 | 21.04 | 39.00 | 1.15 |

**Appendix Table S3. Inferred values for the death model parameters for all 37 OECD countries.**

| Country | $L_d$ | $t_d^0$ | $k_d$ | Pearson $r^2$ | $P\ value$ |
|---|---|---|---|---|---|
| Australia | 79.820 | 0.116 | 99.764 | 0.997 | 3E-120 |
| Austria | 79.467 | 0.141 | 616.932 | 0.999 | 5E-151 |
| Belgium | 83.543 | 0.156 | 8436.879 | 0.999 | 3E-156 |
| Canada | 96.174 | 0.127 | 5801.720 | 0.999 | 5E-161 |
| Chile | 92.790 | 0.117 | 347.588 | 0.999 | 5E-145 |
| Colombia | 97.281 | 0.103 | 572.346 | 0.997 | 8E-125 |
| Czech Republic | 81.227 | 0.150 | 261.615 | 0.998 | 4E-128 |
| Denmark | 80.952 | 0.123 | 518.233 | 0.998 | 1E-131 |
| Estonia | 81.765 | 0.149 | 56.672 | 0.997 | 3E-123 |
| Finland | 91.075 | 0.144 | 284.921 | 0.998 | 5E-133 |
| France | 79.648 | 0.152 | 25804.115 | 0.999 | 1E-154 |
| Germany | 84.526 | 0.132 | 7663.329 | 0.999 | 2E-153 |
| Greece | 75.937 | 0.124 | 147.618 | 0.998 | 3E-132 |
| Hungary | 90.482 | 0.133 | 427.210 | 0.999 | 1E-152 |
| Iceland | 74.742 | 0.160 | 10.279 | 0.991 | 1E-96 |
| Ireland | 90.177 | 0.144 | 1554.827 | 0.999 | 2E-149 |
| Israel | 82.942 | 0.153 | 244.299 | 0.999 | 2E-151 |
| Italy | 73.756 | 0.108 | 29872.992 | 0.998 | 5E-137 |
| Japan | 98.634 | 0.091 | 945.204 | 0.999 | 1E-156 |
| Latvia | 91.404 | 0.157 | 19.387 | 0.995 | 1E-111 |
| Lithuania | 81.545 | 0.141 | 49.072 | 0.999 | 6E-145 |
| Luxembourg | 77.771 | 0.134 | 97.869 | 0.998 | 2E-135 |
| Mexico | 105.667 | 0.115 | 5756.191 | 0.999 | 5E-146 |
| Netherlands | 81.157 | 0.122 | 5434.047 | 0.999 | 3E-141 |
| New Zealand | 86.538 | 0.228 | 20.943 | 0.998 | 1E-127 |
| Norway | 78.999 | 0.155 | 218.777 | 1.000 | 1E-171 |
| Poland | 90.445 | 0.123 | 860.312 | 0.999 | 5E-151 |
| Portugal | 83.744 | 0.123 | 1153.739 | 0.999 | 6E-148 |
| Slovakia | 90.344 | 0.197 | 26.611 | 0.997 | 2E-122 |
| Slovenia | 81.029 | 0.142 | 99.367 | 0.999 | 2E-141 |
| South Korea | 63.564 | 0.093 | 257.983 | 0.999 | 3E-157 |
| Spain | 75.379 | 0.133 | 25559.955 | 0.998 | 1E-132 |
| Sweden | 86.054 | 0.116 | 3732.794 | 0.999 | 4E-138 |
| Switzerland | 78.156 | 0.137 | 1822.825 | 0.999 | 6E-153 |
| Turkey | 87.634 | 0.134 | 3915.683 | 1.000 | 1E-166 |
| United Kingdom | 85.270 | 0.132 | 31693.420 | 0.999 | 1E-147 |
| United States | 88.943 | 0.119 | 164398.018 | 0.999 | 2E-144 |
| **Mean** | **84.556** | **0.136** | **8886.042** | **0.998** | |
| **std** | **7.952** | **0.025** | **27305.588** | **0.001** | |

**Appendix Table S4. Regression analysis.**

| Country | Population size | $L_d$ | $log_{10}\left(\frac{L_d}{pop\ size}\right)$ | Ten deaths day (relative to Jan-13) | Social distancing start time | $\tau$ |
|---|---|---|---|---|---|---|
| Australia | 25499884 | 99.76 | -5.41 | 73 | 54.56 | -18.44 |
| Austria | 9006398 | 616.93 | -4.16 | 69 | 54.90 | -14.10 |
| Belgium | 11589623 | 8436.88 | -3.14 | 64 | 55.34 | -8.66 |
| Canada | 37742154 | 5801.72 | -3.81 | 67 | 55.76 | -11.24 |
| Chile | 19116201 | 347.59 | -4.74 | 78 | 50.96 | -27.04 |
| Colombia | 50882891 | 572.35 | -4.95 | 76 | 52.59 | -23.41 |
| Czech Republic | 10708981 | 261.62 | -4.61 | 75 | 55.38 | -19.62 |
| Denmark | 5792202 | 518.23 | -4.05 | 68 | 56.10 | -11.90 |
| Estonia | 1326535 | 56.67 | -4.37 | 80 | 55.05 | -24.95 |
| Finland | 5540720 | 284.92 | -4.29 | 76 | 54.19 | -21.81 |
| France | 65273511 | 25804.12 | -3.40 | 54 | 56.20 | 2.20 |
| Germany | 83783942 | 7663.33 | -4.04 | 62 | 54.39 | -7.61 |
| Greece | 10423054 | 147.62 | -4.85 | 68 | 53.68 | -14.32 |
| Hungary | 9660351 | 427.21 | -4.35 | 72 | 55.22 | -16.78 |
| Iceland | 341243 | 10.28 | -4.52 | 98 | 57.48 | -40.52 |
| Ireland | 4937786 | 1554.83 | -3.50 | 73 | 52.61 | -20.39 |
| Israel | 8655535 | 244.30 | -4.55 | 74 | 54.17 | -19.83 |
| Italy | 60461826 | 29872.99 | -3.31 | 43 | 43.95 | 0.95 |
| Japan | 126476461 | 945.20 | -5.13 | 56 | 74.16 | 18.16 |
| Latvia | 1886198 | 19.39 | -4.99 | 100 | 54.66 | -45.34 |
| Lithuania | 2722289 | 49.07 | -4.74 | 82 | 56.19 | -25.81 |
| Luxembourg | 625978 | 97.87 | -3.81 | 74 | 55.27 | -18.73 |
| Mexico | 128932753 | 5756.19 | -4.35 | 74 | 59.86 | -14.14 |
| Netherlands | 17134872 | 5434.05 | -3.50 | 60 | 55.47 | -4.53 |
| New Zealand | 4822233 | 20.94 | -5.36 | 95 | 57.79 | -37.21 |
| Norway | 5421241 | 218.78 | -4.39 | 70 | 54.42 | -15.58 |
| Poland | 37846611 | 860.31 | -4.64 | 71 | 53.57 | -17.43 |
| Portugal | 10196709 | 1153.74 | -3.95 | 68 | 55.16 | -12.84 |
| Slovakia | 5459642 | 26.61 | -5.31 | 96 | 53.11 | -42.89 |
| Slovenia | 2078938 | 99.37 | -4.32 | 76 | 52.68 | -23.32 |
| South Korea | 51269185 | 257.98 | -5.30 | 43 | -0.78 | -43.78 |
| Spain | 46754778 | 25559.96 | -3.26 | 54 | 55.52 | 1.52 |
| Sweden | 10099265 | 3732.79 | -3.43 | 64 | 53.94 | -10.06 |
| Switzerland | 8654622 | 1822.82 | -3.68 | 60 | 52.48 | -7.52 |
| Turkey | 84339067 | 3915.68 | -4.33 | 69 | 58.83 | -10.17 |
| United Kingdom | 67886011 | 31693.42 | -3.33 | 60 | 55.20 | -4.80 |
| United States | 331002651 | 164398.02 | -3.30 | 49 | 57.78 | 8.78 |
