# Supplementary material for: COVID‐19 pandemic‐related lockdown: response time is more important than its strictness
Source: EMBO Mol Med. 2020 Oct 19;12(11):e13171. doi: 10.15252/emmm.202013171 (PMC7645374; doi:10.15252/emmm.202013171)
Supplement: Supplementary file 2 — Expanded View Figures PDF [file EMMM-12-e13171-s002.pdf]

Expanded View Figures

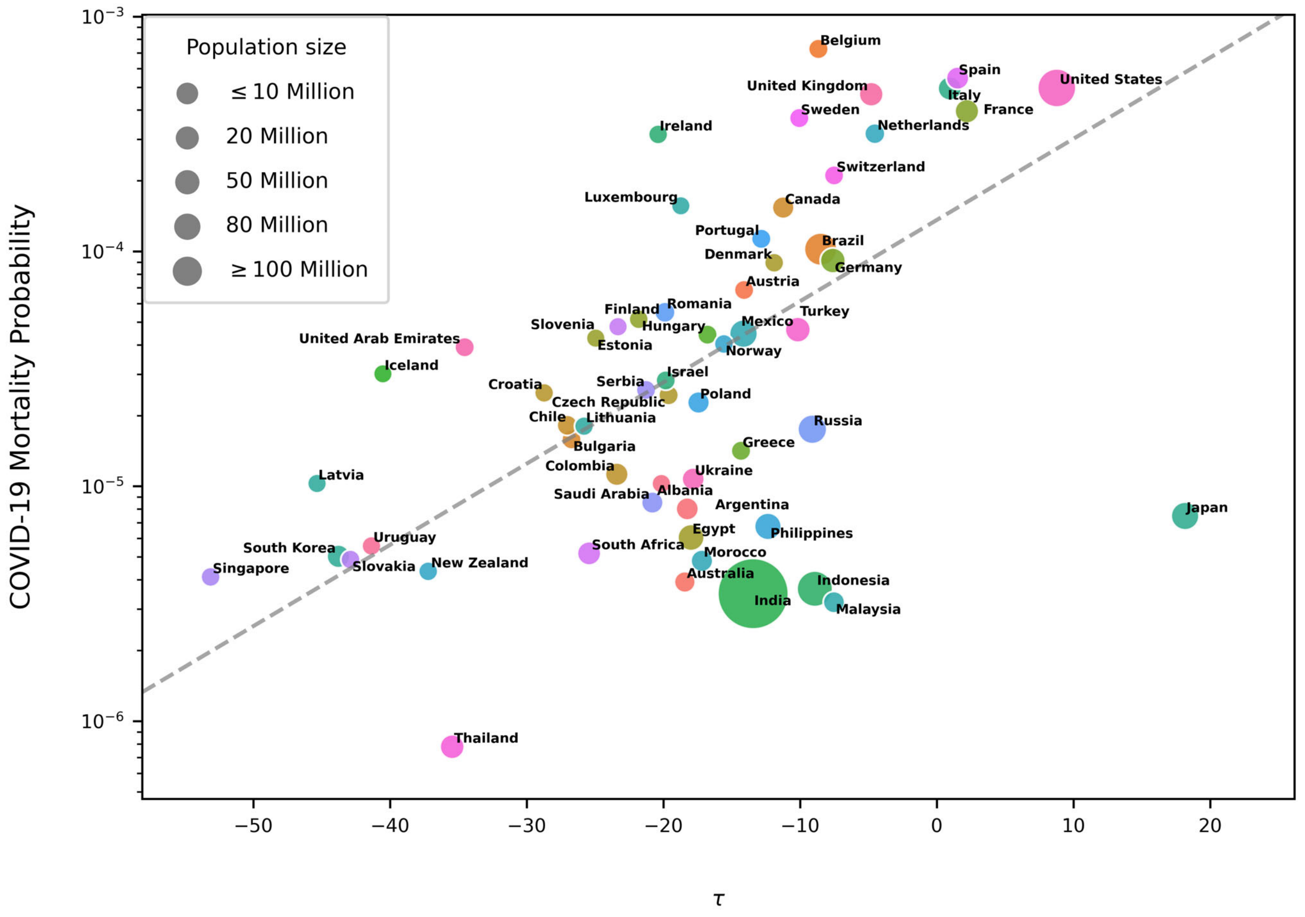

Figure EV1. A semi-logarithmic scatter plot of the *COVID-19 Mortality Probability* and τ in 58 countries.

The *x*-axis represents τ, the difference between the *social distancing start time* and the day in which the first ten deaths were recorded for the respective country (intuitively, the response time). The *y*-axis represents the *COVID-19 Mortality Probability* in a logarithmic scale. Among the 63 for which mobility and death data exist, Cambodia, Hong Kong, Vietnam, Taiwan, and Macau did not reach ten deaths before May 10. Dot sizes are proportional to population sizes. Correlation $r^2 = 0.28$ (P value = 2e-5) when including Japan and $r^2 = 0.37$ (P value = 4e-7) excluding Japan. The dashed line corresponds to the fitted regression, excluding Japan log (COVID 19 Mortality Probability) = 0.035τ –3.89.

© 2020 The Authors

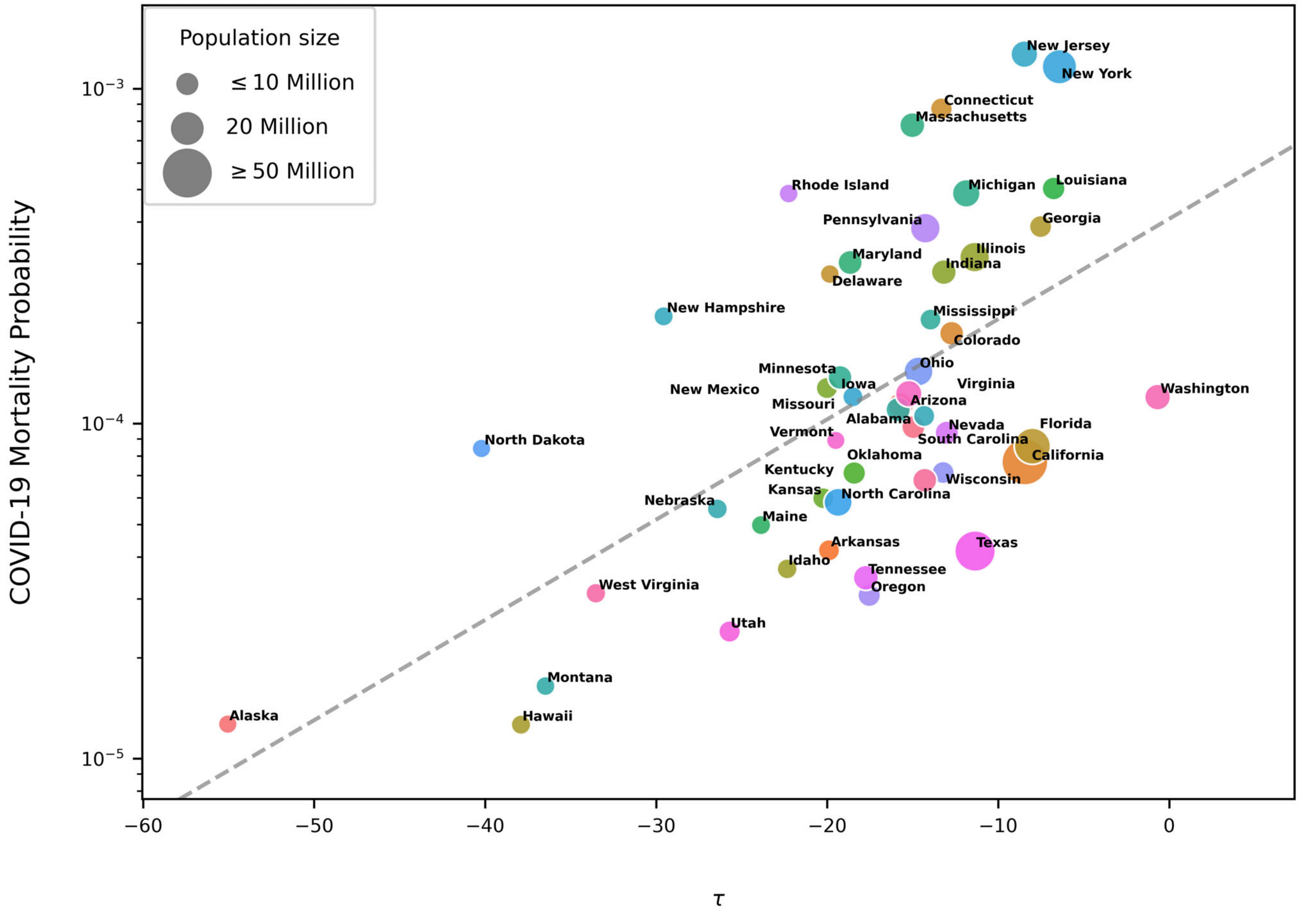

Figure EV2. A semi-logarithmic scatter plot of the *COVID-19 Mortality Probability* and τ for states within the United States.

The *x*-axis represents τ, the difference between the *social distancing start time* and the day in which the first ten deaths was recorded for the respective country (intuitively, the response time). The *y*-axis represents the *COVID-19 Mortality Probability* in a logarithmic scale. Dot sizes are proportional to population sizes. Pearson $r^2 = 0.36$ (*P* values = 8e-6). Wyoming and South Dakota were excluded due to insufficient data. The dashed line corresponds to the fitted regression, log (COVID 19 Mortality Probability) = 0.03τ–3.39.

 © 2020 The Authors

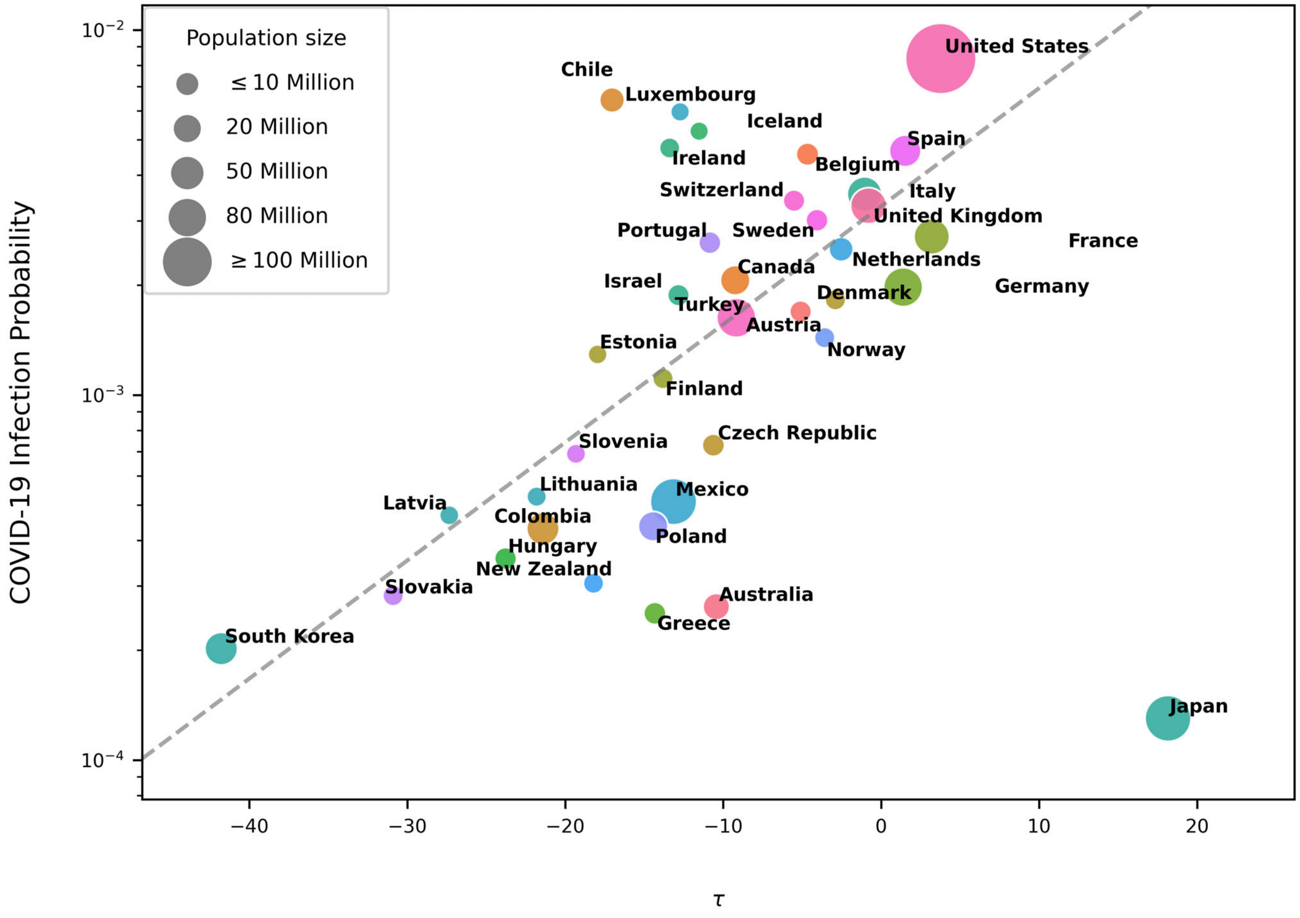

Figure EV3. A semi-logarithmic scatter plot of the *COVID-19 Infection Probability* and τ.

The *x*-axis represents τ, the difference between the *social distancing start time* and the day in which the first 500 confirmed cases were recorded for the respective country (intuitively, the response time). The *y*-axis represents the *COVID-19 Infection Probability*, which was computed by fitting a logistic function to the daily confirmed cases, similar to the *COVID-19 Mortality Probability* (in a logarithmic scale). Dot sizes are proportional to population sizes. Pearson $r^2 = 0.18$ (*P* value 8e-3) when including Japan and $r^2 = 0.47$ (*P* value 4e-6) excluding Japan. The dashed line corresponds to the fitted regression, excluding Japan, log(COVID 19 Infection Probability) = 0.032τ–2.48.

© 2020 The Authors

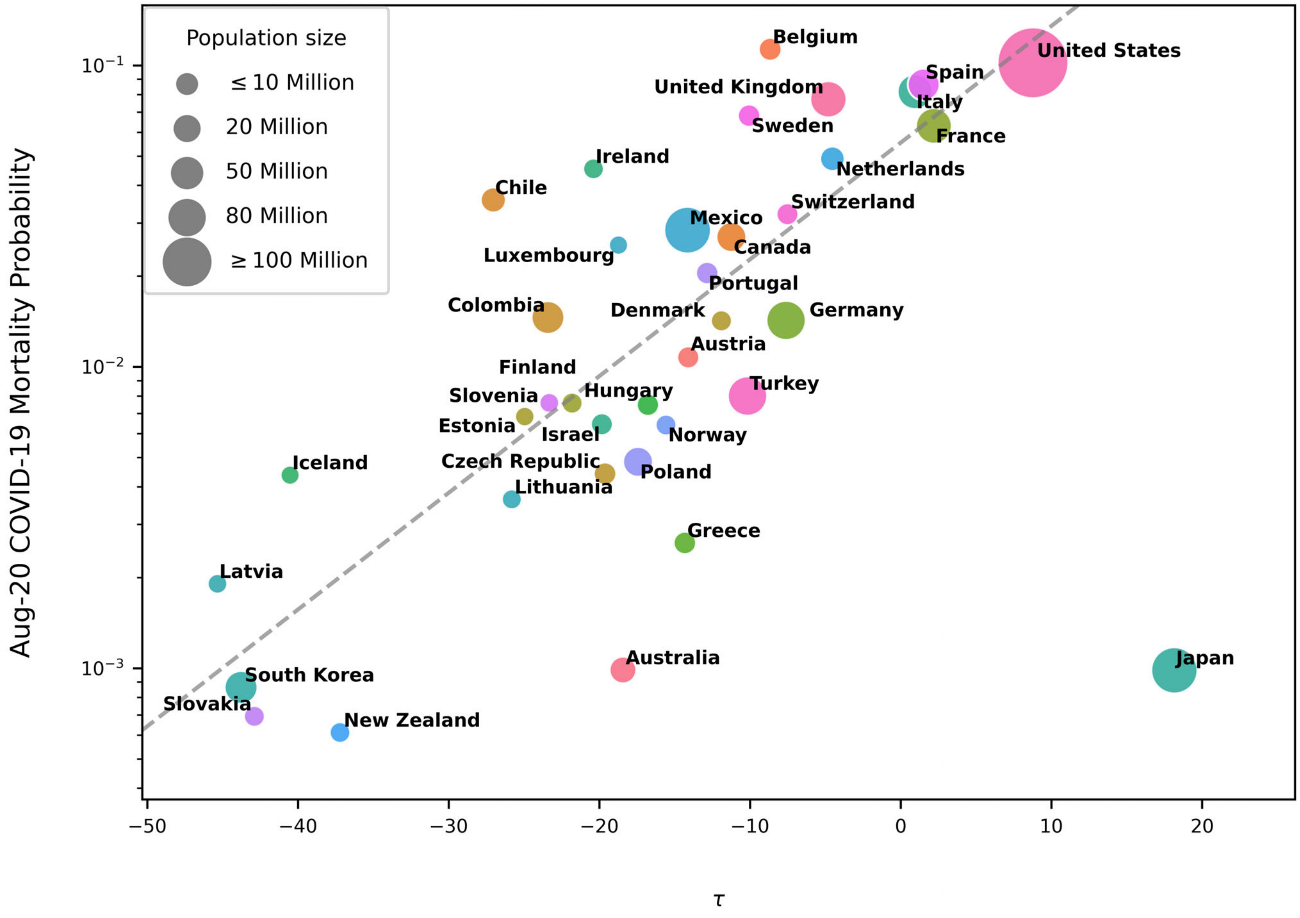

Figure EV4. A semi-logarithmic scatter plot of the Aug-20 *COVID-19 Mortality Probability* and τ in the OECD countries.

The *x*-axis represents τ, the difference between the *social distancing start time* and the day in which the first ten deaths were recorded for the respective country (intuitively, the response time). The *y*-axis represents the Aug-20 *COVID-19 Mortality Probability* in a logarithmic scale. Dot sizes are proportional to population sizes. Pearson r^2 = 0.34 (*P* value = 2e-4) when including Japan and r^2 = 0.62 (*P* value = 1e-8) when excluding Japan. The dashed line corresponds to the fitted regression, excluding Japan: log (Aug–20 COVID 19 Mortality Probability) = 0.039τ–1.26.

© 2020 The Authors

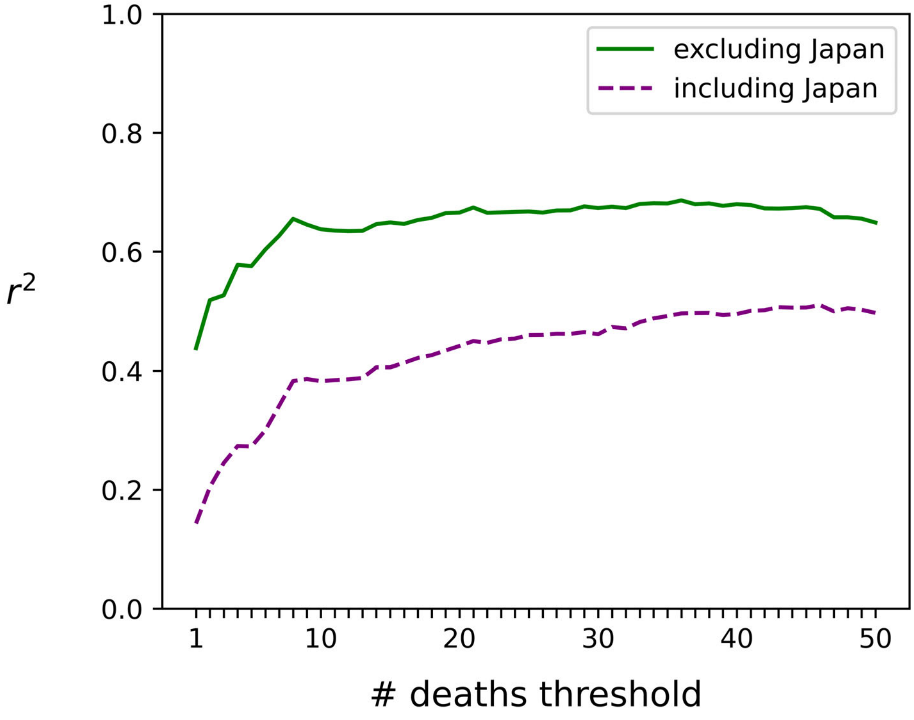

Figure EV5. The correlation between τ and the log *COVID-19 Mortality Probability* for increasing number of deaths as thresholds.

τ was computed as the difference between the day in which increasing number of deaths (*x*-axis) were reported. For each threshold, the correlation was computed between τ and the log *COVID-19 Mortality Probability* across countries, while including or excluding Japan. The correlation was computed over the OECD countries that have sufficient data for each threshold. Iceland, Latvia, New Zealand, and Slovakia reported 10, 19, 22, and 27 deaths overall until May 10; therefore, the correlation was computed for 37 countries from one to 10 deaths, 36 countries from 11 to 18 deaths, 35 countries from 19 to 21 deaths, 34 countries from 22 to 26 deaths, and for 33 countries from 27 to 50 deaths.

© 2020 The Authors
